# Contribution of Neuroimaging Studies to Understanding Development of Human Cognitive Brain Functions

**DOI:** 10.3389/fnhum.2016.00464

**Published:** 2016-09-15

**Authors:** Tomoyo Morita, Minoru Asada, Eiichi Naito

**Affiliations:** ^1^Graduate School of Engineering, Osaka UniversitySuita, Japan; ^2^Center for Information and Neural Networks (CiNet), National Institute of Information and Communications Technology (NICT)Suita, Japan; ^3^Graduate Schools of Medicine and Frontier Biosciences, Osaka UniversitySuita, Japan

**Keywords:** development, functional MRI, human brain, neuroimaging, structure, social cognition

## Abstract

Humans experience significant physical and mental changes from birth to adulthood, and a variety of perceptual, cognitive and motor functions mature over the course of approximately 20 years following birth. To deeply understand such developmental processes, merely studying behavioral changes is not sufficient; simultaneous investigation of the development of the brain may lead us to a more comprehensive understanding. Recent advances in noninvasive neuroimaging technologies largely contribute to this understanding. Here, it is very important to consider the development of the brain from the perspectives of “structure” and “function” because both structure and function of the human brain mature slowly. In this review, we first discuss the process of structural brain development, i.e., how the structure of the brain, which is crucial when discussing functional brain development, changes with age. Second, we introduce some representative studies and the latest studies related to the functional development of the brain, particularly for visual, facial recognition, and social cognition functions, all of which are important for humans. Finally, we summarize how brain science can contribute to developmental study and discuss the challenges that neuroimaging should address in the future.

## Introduction: The Field of Developmental Cognitive Neuroscience

Humans undergo significant physical and mental changes from birth to adulthood. Neonates have the lowest ability to survive, but various abilities related to perceptual, cognitive and motor functions mature over the course of approximately 20 years after birth. We believe that merely studying behavioral changes is not sufficient to fully elucidate the developmental processes of these different functions. Deeper and more comprehensive understanding of the developmental processes of humans will be obtained from understanding the development of the brain. A new approach to understanding the development of the brain has recently emerged; a scientific field termed developmental cognitive neuroscience came into being in the late 1990s (Johnson, 1997). Since then, this scientific field has been steadily growing (Munakata et al., 2004; Blakemore, 2012). Around the time of this field’s emergence, there was dramatic progress in noninvasive neuroimaging technology, which enabled the measurement and imaging of brain activity without injuring the brain while an individual was engaged in mental and/or physical activities.

Neuroimaging techniques are largely classified into the following two categories: one is the technique to measure the electric activity of cell groups in the brain, such as electroencephalography (EEG) and magnetoencephalography (MEG), and the other is the technique to measure the change in blood flow associated with brain activity, such as functional magnetic resonance imaging (fMRI), near-infrared spectroscopy (NIRS), and positron emission tomography (PET). Because the characteristics of these techniques differ from each other, the appropriate technique should be chosen depending on the object or content to be measured.

EEG is a technique used to measure electric activity in the brain using electrodes attached to the scalp. The response of the brain to an object or a stimulus is obtained by averaging the transiently produced brain potentials associated with a specific event; these are called event-related potentials (ERPs). Because EEG can measure brain activity with high temporal precision (high temporal resolution), it is suitable for capturing temporal changes in brain activity. On the other hand, its drawback is that it cannot identify the site of activity with high precision (low spatial resolution). This technique also has other problems, such as difficulty measuring deep brain activity with high precision.

MEG, another technique used to measure electric activity, can more accurately measure the change of magnetic field produced by electric activity. In contrast to the electric field, the magnetic field is less likely to be affected by resistance due to the skull or the scalp; therefore, MEG can attain higher spatial resolution than EEG. On the other hand, compared to EEG, MEG has drawbacks in that the instrument is larger and is more likely to be affected by noise caused by the body movement of a participant.

The most notable characteristic of MRI is that it can visualize the structural images of the brain (structural MRI, diffusion MRI), which are not possible with EEG or MEG. Structural MRI provides information to describe the shape, size, and integrity of gray and white matter structures in the brain. On the other hand, diffusion tensor imaging (DTI) can generate images of the fiber structure (fiber orientation and size) of the white matter, and the maturity of the white matter, including the degree of myelination, can be quantified. In addition to the structural images of the brain, the functional images of the brain can be also visualized by using the MRI. This technology, termed fMRI, can measure the blood oxygenation level-dependent (BOLD) signal, which is well correlated with the local field potential (synaptic activity; Logothetis et al., 2001). This technique is inferior to EEG or MEG in terms of temporal resolution, but it is superior in terms of spatial resolution, with precision on the order of a millimeter, and one can very precisely measure the activities in deeper cortical and subcortical brain structures. However, it has some restrictions for measurement, such as that a participant must lie down in an enclosed space and is restricted in body movement. These restrictions make it difficult to conduct fMRI study in children. To circumvent this difficulty, fMRI scans in infants and preschool children have been conducted during natural sleep (Anderson et al., 2001; Dehaene-Lambertz et al., 2002; Wilke et al., 2003; Redcay et al., 2007) or under anesthesia (Yamada et al., 1997; Born et al., 1998; Martin et al., 1999). The fMRI technique can be used not only to identify brain regions active when participants perform a task that requires a particular neuronal process, but also to examine functional connectivity across multiple brain regions while participants are not performing a particular task (usually not in sleep). The latter approach, called resting-state fMRI, may elucidate basic functional networks across multiple brain regions, where neuronal activities synchronize based on spontaneous low frequency fluctuations (<0.1 Hz) in the BOLD signal (Biswal et al., 1995; Fox and Raichle, 2007; Biswal, 2012). Recent resting-state fMRI studies have revealed the existence of basic functional networks in the infant brain (Fransson et al., 2007, 2009) and the maturation of the local-to-distributed organization of functional networks (Fair et al., 2009), both of which are difficult to unveil with behavioral studies. Recently, researchers have started to investigate the resting-state functional connectivity not only by fMRI but also by EEG and NIRS. This approach is particularly effective and useful in infants and younger children because we could learn how the basic functional networks develop in the human brain without requiring them to perform a complex task (Fransson et al., 2007; Homae et al., 2010; Gao et al., 2016).

NIRS is a technique used to measure changes in oxygenated and deoxygenated hemoglobin in specific brain areas in response to various stimuli. The temporal resolution of NIRS is not as high as that of EEG or MEG, and its spatial resolution is lower than that of MRI. However, NIRS has the advantages of being portable, easy to use, quiet, and relatively less sensitive to motion artifacts (Gervain et al., 2011). Therefore, NIRS is suitable for the measurement of brain activity in infants and children. In addition, the layers of the scalp and the skull of an infant are thin, providing a better condition for measuring cerebral blood flow change in the infant brain. Because of these advantages, NIRS has contributed to the understanding of developmental processes during infancy from visual and auditory perception to the acquisition of language (Taga et al., 2003; Minagawa-Kawai et al., 2008; Grossmann et al., 2010; Gervain et al., 2011; Otsuka, 2014).

The above-described techniques have been gradually used to measure brain activity in children since around 2000. However, the number of neuroimaging studies in children has been far less than that in adults. The reason for this is that brain activity measurement entails more restrictions than behavioral observation. For example, movement of the head or body of a participant during brain activity measurement causes noise in the measurement data. Therefore, we usually ask participants not to move their heads or bodies as much as possible. This is relatively easy for adults, but it is very difficult for children who naturally want to move. Therefore, brain activity measurement in children requires more detailed considerations concerning experimental designs such as scanning time, the content of tasks, and the experimental environment compared with that in adults (Slifer, 1996; Slifer et al., 2002; de Bie et al., 2010; Raschle et al., 2012). Thus, experiments in children require much more time and effort than those in adults.

Since the advent of developmental cognitive neuroscience, neuroimaging studies in infants and preschool children have attracted the interest of many researchers. This may be because there are observable, dramatic behavioral changes during the infancy and preschool period, and we may therefore assume drastic changes occur in the brain as well. On the other hand, it can be said that studies in school-age and older children have attracted less interest. One reason is that the amount of daily activities enormously increases in school-age and older children when compared with infants and preschool children. The dramatic advance of cognitive abilities also becomes less prominent, and some of the cognitive functions are becoming settled (e.g., Theory of Mind [ToM]; Wellman et al., 2001) and comparable with adults (e.g., Wisconsin Card Sorting Test; Chelune and Baer, 1986; Welsh et al., 1991) in school-age and older children. However, even in these children, there are several pieces of evidence showing that the brains of school-age, pubescent and adolescent children who seemingly behave like adults are still developing. This is because the structure of the whole brain grows slowly over 20 years or longer (Giedd et al., 1999; Gogtay et al., 2004), and the functions of the brain are also thought to mature slowly (Blakemore and Choudhury, 2006; Casey et al., 2008). As will be introduced in Section “Functional Development of the Brain”, previous studies have suggested that there is a significant difference between how children use the brain and how adults use the brain even though no large differences are found at the behavioral level (e.g., Wang et al., 2006; Blakemore et al., 2007; Scherf et al., 2007).

In favor of this view, we have recently shown that primary school children at the age of 8–11 years old can precisely recognize their own faces, as these are distinct from others’ faces just like adults. However, when we examined the brain activity with fMRI, we discovered that the activation patterns were substantially different from those observed in adults (Morita et al., 2016). This suggests that the subjective experience when children recognize their own faces is different from that of adults. This fMRI finding may predict distinguishing qualia of bodily self-awareness in children at these ages, which could be carefully evaluated by behavioral investigations.

Another of our fMRI studies showed that 8–11-year-old children can perform hand movements at 1 Hz just like adults, but the functional connectivity between the motor cortex and the cerebellum during the movements is significantly weaker in children when compared with adults (Naito et al., 2016). If we consider the general notion of the importance of the cerebellum in timing motor control and learning, which is established fundamentally by studies in adults (Ivry, 1996), our neuroimaging result leads us to hypothesize that there must be a qualitative behavioral difference in timing motor control and learning between children and adults and motivates us to investigate in our future studies.

Hence, neuroimaging investigation of how the human brain develops can not only provide valuable knowledge about human development, which is difficult to obtain from behavioral studies, but also provide new hypotheses and models, which should be tested in behavioral studies. In addition, parallel investigation into the development of a certain brain function and its underlying neuronal mechanisms may promote our understanding about key brain structures and networks that implement the function in the mature adult brain and about how these structures and networks acquire this function.

With this background information, we will first outline the process of structural development of the brain, which can be a basis for functional development of the brain. Second, we will introduce some representative studies and the latest studies related to the functional development of the brain, particularly for visual, facial recognition, and social cognition functions. We deal with these functions because they are important for humans, who lead social lives. Finally, we will summarize how brain science can contribute to developmental study in general and discuss the challenges that neuroimaging should address for the future.

## Developmental Changes Observed in Brain Structure

### Two Different Dynamics in the Development of Brain Structure

**Figure 1:**
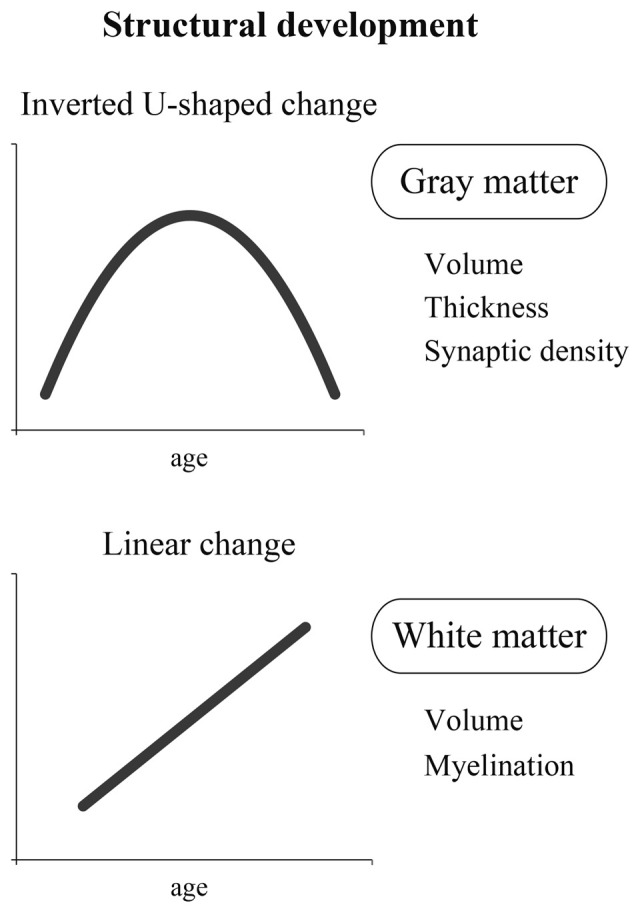
**A schema of two different dynamics of structural development: inverted U-shaped change and linear change.** The former is represented by the change in gray matter containing cell bodies that process information. The latter is represented by the change in white matter containing nerve fibers that transmit information.

As described previously, it is essential to study both structure and function to clarify the developmental process of the brain. Here, we first discuss the structural development of the brain. In the literature, it seems that structural changes in the brain are largely classified into two categories. One is an inverted U-shaped change, and the other is a linear change (Figure 1).

#### Inverted U-Shaped Change

A representative inverted U-shaped developmental change is observed in the synaptic connection. An important finding regarding synaptic connections was made before neuroimaging technology became available. An inverted U-shaped developmental pattern that includes a phase of rapid increase in synapse density followed by a phase of synapse-elimination has been found in non-human brains, such as kitten and monkey (Cragg, 1975; Lund et al., 1977; Rakic et al., 1986; Bourgeois and Rakic, 1993). In human research, a pioneering study was done by Huttenlocher who carefully examined the synapses in postmortem human brains and investigated synaptic density changes according to the stages of brain development. This study demonstrated the pattern of a series of changes: synaptic density begins to increase after birth, followed by a rapid increase during a certain time period until it peaks; then, it begins to decrease and reaches the density level of adults (Huttenlocher, 1979; Huttenlocher et al., 1982). In other words, synapses are excessively formed for a brief time, but the excessive, unnecessary synapses are pruned afterwards. Interestingly, the temporal change of synaptic density differs depending on the region of the brain (Huttenlocher and Dabholkar, 1997). For example, in the visual cortex, synaptic density is reported to rapidly increase at the age of 2–3 months, peak at 4–12 months, and then decrease to the level of adults at the age of 2–4 years. In contrast, in the prefrontal cortex, all of the above timings are delayed, and it is reported that the adult density level is achieved at the age of approximately 15–20 years. Recently, similar inverted U-shaped patterns have been reported when researchers measured the synaptic marker proteins (Glantz et al., 2007) and the synaptic spine density (Petanjek et al., 2011) in the prefrontal cortices of postmortem human brains. All of this evidence suggests that the developmental curve of synapse density shows an inverted U-shape in humans.

In favor of this view, neuroimaging studies have also demonstrated that the volume and the thickness of the gray matter show an inverted U-shaped curve in the development of human cerebral cortices (Jernigan et al., 1991; Giedd et al., 1999; Gogtay et al., 2004). During 1–2 years after birth, when synaptic density rapidly increases (Huttenlocher, 1979; Huttenlocher et al., 1982), cortical gray matter volume also rapidly increases (Knickmeyer et al., 2008; Gilmore et al., 2012). In addition, the peak timing of an inverted U-shape in the development of gray matter volume varies across cortical regions (Giedd et al., 1999; Gogtay et al., 2004), just like that in the development of synaptic density (see above and Huttenlocher and Dabholkar, 1997). This collection of evidence suggests that the morphological change (i.e., inverted U-shape) in the gray matter reflects, at least in part, the excessive formation of synapses and the subsequent pruning.

#### Linear Change

In contrast to the inverted U-shaped change discussed above, it is generally believed that myelin shows a linear developmental change (Yakovlev and LeCours, 1967; Benes, 1989). Myelin is an insulator that is formed around the axon of a nerve fiber, and the presence of myelin makes efficient signal transmission possible. In other words, the brain with advanced myelination is considered to be a mature brain that can process information efficiently and at a high speed. It is reported that there is little myelination in the brain of a neonate soon after birth. Therefore, efficient and high-speed information processing is hardly possible in the brain of a neonate.

Previous studies using DTI (see “Introduction: The Field of Developmental Cognitive Neuroscience” Section) showed that the white matter matures linearly and that the maturation process continues until the age of 20–30 years (Klingberg et al., 1999; Barnea-Goraly et al., 2005). These results are in good agreement with the findings of the developmental change of myelination observed in the examination of postmortem brains before the development of the DTI technique (Yakovlev and LeCours, 1967; Benes, 1989). As described above, the developmental curve of myelination gradually ascends with time. Just as the volume of the gray matter fits an inverted U-shaped curve corresponding with synaptic density, the volume of the white matter (which contains abundant myelin) fits a linear curve corresponding with myelination. The volume of the white matter increases nearly linearly with age, independent of brain region, and this increase is known to continue until the age of approximately 20 years old (Giedd et al., 1999; Paus et al., 2001; Sowell et al., 2002; Lebel et al., 2008).

In sum, the developmental change of the human brain seems to show at least two different dynamics: an inverted U-shaped curve and a linear curve. The former can be represented by the change in brain cell bodies that process information, and the latter may be represented by the change in nerve fibers that transmit information by connecting different areas of the brain. However, as recently shown by Shaw et al. (2008), some cortical regions likely show linear changes in the development of cortical thickness. Thus, further neuroimaging studies are definitely needed to fully understand the structural development of the human brain.

### Relationship Between Brain Structure and Cognitive Function

In the previous section, we gave a general view of the structural changes in terms of brain development. In this section, we first introduce evidence in the adult brain showing how the brain changes its structures in association with the development of particular cognitive functions, and we will then discuss the relationship between brain structures and cognitive functions from the developmental viewpoint.

#### Adult Brain

In the adult brain, it has been shown that particular regions of the brain are enlarged or diminished depending on the degree of the trait or the ability of the individual (Kanai and Rees, 2011). For example, in the case of a technician who has mastered a certain skill over a long period of time, the volume of the part of the brain associated with that particular skill is increased in that adult’s brain (taxi driver: Maguire et al., 2000; pianist: Gaser and Schlaug, 2003). These are examples of what happens during the process of mastering certain skills over a long period of time.

The structural changes of the brain can also be observed in ordinary people. DeYoung et al. (2010) used a personality test developed by Goldberg (1990) called the Big Five Test in order to investigate the relationship between the five elements that form personality (extroversion, agreeableness, conscientiousness, neuroticism and openness to experience) and the size of different brain regions. They found the following changes: persons with higher extroversion have a larger medial orbitofrontal cortex, which is important for reward processing, and persons with higher neuroticism have a larger amygdala and cingulate cortex, which are involved in the processing of negative emotion (DeYoung et al., 2010).

#### Developmental Viewpoint

The two types of developmental changes in brain structure (both inverted U-shaped change and linear change) seem to be deeply associated with development of cognitive functions.

##### Inverted U-shaped change

Shaw et al. (2006) showed a relationship between an inverted U-shaped change in the gray matter and cognitive function in children and adolescents. In this study, the thickness of the cerebral cortex of the brains of approximately 300 healthy children and adults was calculated using structural MRI. At the same time, an IQ test, which measures general intelligence level, was conducted. Participants were grouped into three categories based on their scores, i.e., the “superior group,” the “high group,” and the “average group.” The result clearly showed that the patterns of developmental changes observed in the thickness of the cortex of the frontal region were quite different among the groups. In the superior group, the cortex was relatively thin at first, increasing rapidly to reach a peak at the age of approximately 11 years, and then rapidly thinning again. Thus, this group showed rapid and large inverted U-shaped change in the gray matter thickness of the frontal cortex. Compared to the superior group, the change of the cortical thickness was relatively slow and small in the average group, and the thickness reached a peak earlier at the age of 7–8 years (Shaw et al., 2006). Hence, how the gray matter thickness of the frontal cortex changes during childhood appears to affect intellectual level.

Some evidence shows that the marked development of a cognitive function occurs coincidently with the period of thinning in a certain cortical area (Casey et al., 2005; Tau and Peterson, 2010). For example, an increase in language vocabulary is associated with cortical thinning in the left dorsolateral frontal and lateral parietal regions at the age of 5–11 years (Sowell et al., 2004). In addition, improvement of hand motor skill is associated with cortical thinning in the left primary motor cortex (M1) during the same age period (Lu et al., 2007). Finally, improvement of cognitive control ability seems to be related to cortical thinning in the anterior cingulate cortex and the right inferior frontal gyrus at the age of 5–10 years (Kharitonova et al., 2013).

As discussed above (in Section “Developmental Changes Observed in Brain Structure”, “Inverted U-Shaped Change”), the inverted U-shaped change in the gray matter reflects, at least in part, the excessive formation of synapses and subsequent pruning, and this series of the synaptic changes likely occurs in several brain regions in addition to the frontal cortex (Huttenlocher, 1979; Huttenlocher et al., 1982). Hence, we may assume that the formation and pruning of synaptic connections during childhood is the key to build efficient neural circuits in the cortex, leading to its excellent functioning, and marked development of a cognitive function may occur coincidently with the period of synaptic pruning.

##### Linear change

A growing body of literature also shows the relationship between linear structural change in white matter and cognitive performance during development. For example, high anisotropy measured by DTI that reflects directionality or non-randomness of diffusion in the superior fronto-parietal cortices correlates well with memory capacity (Nagy et al., 2004). In addition, higher anisotropy in the left temporal and parietal lobules correlates well with reading ability (Nagy et al., 2004; Deutsch et al., 2005), and that in the frontal and occipitoparietal association areas is well associated with IQ (Schmithorst et al., 2005). These studies investigated the age-related developmental changes of white matter fibers, which are associated with the development of cognitive abilities. However, one should always bear in mind that these age-related changes also include the effects of individual differences. The best way to purely evaluate age-related change is to conduct a longitudinal cohort study, in which one can follow the change across time in the same individuals (e.g., Yeatman et al., 2012). Altogether, the research described above strongly indicates that developmental changes in the brain structure (both inverted U-shaped change and linear change) are deeply associated with development of cognitive functions.

## Functional Development of the Brain

In the previous section, we outlined two major structural developmental changes of the brain, inverted U-shaped change and linear change, both of which are associated with the development of cognitive functions in general. In this section, among various brain functions, we focus on studies regarding visual, facial recognition, and social cognitive functions, which are very important for humans, who lead social lives.

### Development of Visual Function During Infancy

Neonates have blurred vision immediately after birth, and their decimal visual acuity is considered to be about 40 times worse than that of visually normal adults (Miranda, 1970; Mayer and Dobson, 1982; van Hof-van Duin and Mohn, 1986). Their visual acuity improves rapidly in the first 6 months after birth, and then improves more gradually, reaching the adult level at the ages of 4–6-years-old (Mayer and Dobson, 1982; Courage and Adams, 1990; Ellemberg et al., 1999). In addition to visual acuity, various visual functions, such as color perception and depth perception, develop greatly by the time of preschool. The undeveloped visual functions in infants are likely associated with the immature structure and function of the eye, which receives visual information, and of the brain, which processes visual information and has not sufficiently developed.

Visual information sent from the eye is transferred to the cortical region, called the visual cortex, located at the rear end of the cerebrum. More than 50 years ago, Wiesel and Hubel (1963) demonstrated through animal experiments that the experience of seeing things is very important for the functions of the visual cortex to develop normally. In animals, they showed that visual deprivation at a particular period after birth severely affects the development of normal vision. This experiment demonstrated the presence of a “critical period,” during which the brain is susceptible to plastic change due to environment and experience. In humans, based on the above evidence, we think that the period several years after birth when visual functions enormously develop could be a “sensitive and important period.” During this period, we assume that the function of the visual cortex changes plastically depending on visual experiences.

Here, we introduce a study that successfully captured, by imaging, a functional change occurring in the visual cortices of infants in whom plastic changes are ongoing (Yamada et al., 1997). In this study, a flashing light stimulation was presented to infants aged 1 year or younger, and the response of the visual cortex to the photic stimulation was investigated with fMRI. The results showed that fMRI (BOLD) signals increased in response to the photic stimulation in infants less than 8 weeks of age (corrected for gestational age at birth). The pattern of these signals was the same as that seen in adults. Surprisingly, however, the BOLD signals decreased (negative BOLD) in response to the photic stimulation in infants older than 8 weeks of corrected age (Yamada et al., 1997).

In the adult brain, when neurons fire, local consumption of oxygen increases, and this oxygen consumption in the local blood flow leads to a transient increase of deoxygenated hemoglobin (deoxy-Hb) in the blood. However, as the blood flow continuously supplies, the amount of oxygen supplied eventually exceeds the amount of oxygen consumed. This can wash a way the transiently increased deoxy-Hb so as to decrease its concentration, which enhances the (positive) BOLD signal. In contrast, negative BOLD observed in the infant’s (older than 8 weeks) brain indicates an increase of local deoxy-Hb in response to the photic stimulation.

Exact neuronal mechanisms remain to be unveiled. However, one possible explanation is as follows. The reversing pattern (negative BOLD) can be observed after approximately 2–3 months after birth when the synaptic density (Huttenlocher et al., 1982) and metabolic activities (Chugani and Phelps, 1986) drastically increase in the visual cortex. This suggests the possibility that the regional oxygen demand in infants is greater than that in adults (Yamada et al., 1997; Born et al., 1998; Morita et al., 2000) even when the brain receives the same amount of photic stimulation. Thus, the infant’s brain should require more oxygen than can be supplied by oxygen delivery. This may cause an imbalance between oxygen demand and supply, resulting in an increase of local deoxy-Hb, which is associated with the negative BOLD phenomenon. However, since a different view also exists (Kozberg et al., 2013), further investigations are needed to verify our hypothesis.

Although these studies (Yamada et al., 1997; Born et al., 1998; Morita et al., 2000) were conducted when the arousal state of the tested children was low (under anesthesia), the possibility that this particular condition caused the reverse (negative BOLD) phenomenon has been ruled out. Watanabe et al. (2012) presented a light stimulation to an *awake* 6-month-old infant and measured the activity of the visual cortex in response to the stimulation. The result showed that the amount of oxygenated hemoglobin in the visual cortex significantly decreased during light stimulation. This clearly demonstrated that the visual cortex of an infant shows opposite blood flow dynamics compared to adults even under awake conditions (Watanabe et al., 2012).

Interestingly, this type of negative BOLD phenomenon seen in the visual cortex in infancy and early childhood (Born et al., 1998; Martin et al., 1999; Redcay et al., 2007) has also been observed in other regions of the cerebral cortex (e.g., auditory cortex: Anderson et al., 2001). Namely, this phenomenon seems to be a physiological change that is inevitable for the development of brain function.

**Figure 2:**
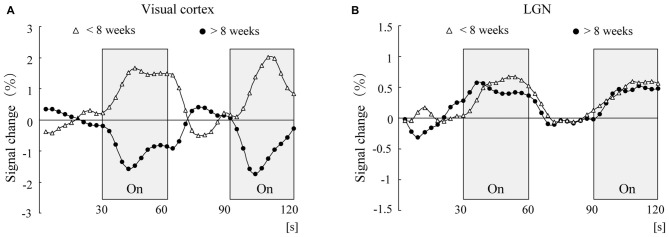
**Time course of adjusted blood oxygenation level-dependent (BOLD) signal changes for the visual cortex (A) and lateral geniculate nucleus (LGN; B) within each group.** The mean values at each scanning time point were obtained for the younger group (open triangles, *n* = 8) and the older group (closed circles, *n* = 8).

This type of negative BOLD response in the visual cortex cannot be seen in the lateral geniculate nucleus (LGN), which is a relay nucleus for the transmission of information from the retina to the visual cortex (Morita et al., 2000). Morita et al. (2000) investigated the LGN activity in response to light stimulation and examined whether the negative BOLD response could also be observed in the LGN. The results showed that while the MRI signal pattern in the visual cortex was reversed at the corrected age of 8 weeks, the MRI signal in the LGN in response to light stimulation was elevated independent of age (Morita et al., 2000; Figure 2). Therefore, negative BOLD response is not observed in the LGN but is observed only in the cerebral visual cortex. This may be related to the fact that the maturation of the LGN is almost completed at the time of birth (see below).

Although rapid developmental changes occur in the human cerebral cortical region after birth, it is reported that in the phylogenetically older cortical regions (such as the LGN), rapid developmental changes occur during the fetal stage. For example, the number of optic tracts that run from the retina to the LGN peaks at the fetal age of 16–17 weeks and then decreases (Provis et al., 1985). In addition, excessive formation of synapses in the LGN is reported to occur at the fetal age of 16–17 weeks (Wadhwa et al., 1988; Khan et al., 1994). Thus, we may understand this as follows: at the time of birth, the LGN has already matured to the same level observed in adults; therefore, the level of MRI signal response to light stimulation in infants is the same as that in adults. The above results strongly suggest that the negative BOLD response observed in the cerebral cortical region is caused by a rapid developmental change in the cerebral cortex, which is specific to infancy and early childhood.

In sum, during the growth stage, the metabolism of the cerebral cortex is greatly changing probably because of the change in synaptic connections. Thus, the change in metabolic activity may be an inevitable phenomenon for brain development.

### Development of Facial Recognition Function from Infancy to School Age

Faces are the most important and most common visual pattern for humans, who lead social lives. Although all faces have parts (such as the eyes, nose and mouth) similarly arranged, we can distinguish the faces of many people and recognize subtle changes in their expressions. How then does the facial recognition function develop? Here, we give an overview of the neuroimaging studies that have investigated the development of facial recognition.

In the adult’s brain, it is known that a negative ERP component (N170) appears in the occipitotemporal cortices approximately 170 ms after the presentation of a face and that this ERP component becomes significantly larger when faces are viewed rather than other objects, such as a chair or car. Thus, the component is considered specific to facial stimulation (Rossion and Jacques, 2008).

In children, de Haan et al. (2002) investigated in which stage of development this face-specific ERP component first appears. ERPs for upright faces of a human and a monkey were recorded in 3-, 6- and 12-month-old children. An ERP component that responded more strongly to a human face than to a monkey face was observed in the occipitotemporal region in children of all ages. However, this component showed a longer latency than the N170 of the adults and was observed approximately 260–290 ms after the presentation of the faces. This component can be considered the counterpart of the N170 in adults because the recorded brain region is the same, even though its latency is longer than that of the ERP in adults (de Haan et al., 2002; Halit et al., 2003).

This type of brain activity specifically evoked by visual stimulation of human face has also been reported in early infancy (Tzourio-Mazoyer et al., 2002; Csibra et al., 2004; de Heering and Rossion, 2015). For example, de Heering and Rossion (2015) reported that the occipitotemporal (face) region of the right hemisphere in 4–6-month-old infants selectively responds to face stimuli but not to object stimuli. In addition, a PET study reported that even in 2-month-old infants, the occipital region of the right hemisphere, which is activated specifically by faces in adults, was more strongly activated when the infant was exposed to a face than when exposed to mere light stimulation (Tzourio-Mazoyer et al., 2002). These findings indicate that, within at least 6 months after birth, humans likely start to process human faces differently from other objects.

However, the more elaborate facial recognition function does not completely mature within the first year of birth; it takes a much longer time to acquire adult-like processing. Indeed, the ERP component of primary school children is reported to still be different in some brain regions from that of adults. The latency of the N170 component of primary school children becomes nearly the same as that of adults. However, the wave pattern of the component appears to be different until children become approximately 11 years old. Until that time, the waveform of the N170 component is not sharp and has two small peaks. In contrast, when children become 12–13 years or older, the waveform becomes similar to that of adults, i.e., it changes to a sharp, single-peak waveform (Taylor et al., 2004; Miki et al., 2015). However, one must also bear in mind that such age-dependent N170 changes could be observed for non-face objects as well (Kuefner et al., 2010).

Some fMRI studies also show that the neural system for facial recognition is not yet fully developed in the school-age period. Scherf et al. (2007) presented short footage showing faces, objects, buildings and navigation scenes to three groups of 5–8-year-old children, 11–14-year-old adolescents, and adults and measured brain activity by fMRI. The results showed similar selective activity in response to objects and places among all groups, but selective responses to faces were significantly different between groups. Namely, in the adult and adolescent groups, multiple brain regions, including the fusiform face area (FFA), showed selective responses to faces, which were not observed in the group of children. This finding indicates that face-selective processing is immature in the brains of children (Scherf et al., 2007). Differences in facial processing between elementary school children and adults are also reported in other fMRI studies (Passarotti et al., 2003; Golarai et al., 2007). Golarai et al. (2007) showed that the volume of the brain region that selectively responds to faces (FFA) was markedly smaller in elementary school children than in adults.

These neuroimaging studies on face recognition suggest that there is a significant difference between how school-age children process faces and how adults process them, even during passive observation without any cognitive load (e.g., memory, decision, etc.). This may provide a possible answer to the debate surrounding the behavioral development of face-specific visual processing. Some researchers proposed that face-specific visual processing is fully mature at around 5 years of age, and the developmental improvement of face processing is due to the maturation of general cognitive abilities such as memory and attention (Crookes and McKone, 2009; McKone et al., 2012). Conversely, other researchers suggested that the developmental improvement in face perception lasts into adolescence or even adulthood (Mondloch et al., 2002; Germine et al., 2011; Susilo et al., 2013; Goffaux et al., 2015). The neuroimaging evidence described here seems to support the latter view of slow developmental changes in facial recognition functions.

In this way, in approximately 1 year after birth, the human brain likely starts to process a human face differently from something that is not a human face. However, this is only the beginning of the development of face recognition. When we consider the above studies (Passarotti et al., 2003; Golarai et al., 2007; Scherf et al., 2007), it seems that more than 10 years of experience seeing faces is required for the FFA to show the selective response to faces. Thus, the brain region that is specialized for face processing emerges along with development. The emergence of such brain region seems to align with the concept of cortical specialization (Johnson, 2001; Cohen Kadosh and Johnson, 2007). One of the main challenges for future neuroimaging studies is the clarification of both structural and functional changes in the brain that are directly associated with the specialization process.

### Development of Social Cognitive Function in the School-Age Period and Beyond

In leading a social life, we frequently experience situations in which we speculate about the feelings of others. The mental activity of speculating about the mental states (intention, emotion, belief, etc.) of others is called “mentalizing” (Frith and Frith, 1999). This mentalizing function is very important in leading a normal social life and interacting with many people; thus, many neuroimaging studies on mentalizing have been conducted in adults.

Although the participants in individual studies are varied, studies in adults who are speculating about another person’s mental states consistently report activity in the medial prefrontal cortex, superior temporal sulcus, temporal parietal junction, temporal pole and other regions; these brain regions are called the social brain (Brothers, 1990; Gallagher and Frith, 2003; Frith and Frith, 2006).

When and how does this mentalizing function develop? In the field of developmental psychology, the Sally-Anne task is often used as a test to examine the presence or absence of mentalizing ability (Baron-Cohen et al., 1985). In this task, children are presented with the following story by using a picture-card show or a puppet show. In this story, there are two characters: Sally and Anne. Sally first hides a toy in a certain place (in a basket), and Anne then moves the toy to a different place (in a box) while Sally is away. After this show, the experimenter asks the child, “Where will Sally go to look for the toy?” Since Sally did not see that Anne moved the toy, she should have the false belief that the toy is still in the basket. If the child correctly understood this false belief, he or she should be able to answer, “In the basket,” but otherwise the child would answer, “In the box.” It is known that most 3-year-olds cannot answer correctly in this task, but the rate of correct answers substantially increases in 5-year-olds, and most primary school children are able to answer correctly (Wellman et al., 2001). Thus, based on this behavioral evidence, the mentalizing ability is believed to be acquired at the age of approximately 4 or 5 years. What is changing in the brain at this age?

Liu et al. (2009) investigated this problem for the first time. They recorded ERPs while 4–6-year-old preschool children performed a false belief task (Liu et al., 2009). A slow negative component (with a latency of approximately 1000 ms) was detected in the left frontal lobe of the preschool children who demonstrated a high rate of correct answers to the false-belief task, also seen in adults; however, this component was not observed in the preschool children with a low rate of correct answers. This research is highly valued as the first study to show a difference in brain activity, according to the presence or absence of mentalizing ability. Unfortunately, however, it could not identify the changes in brain regions directly involved in mentalizing.

Recently, Gweon et al. (2012) reported activity in the mentalizing regions described above, such as the medial frontal cortex and the temporal parietal junction, in preschool-age and elementary school children. The researchers told stories to 5–11-year-old children and adults, depicting: (1) a situation that required understanding the psychological state of others; (2) a situation that required understanding social relations; and (3) a situation of simply physical relations, and simultaneously measured brain activity with fMRI. The results showed that in children at any age, similar to adults, the mentalizing regions were activated when the participants were trying to understand the psychological states of other persons. However, in younger children (from 5 to 8.5 years), the mentalizing regions were equally recruited for all of the above three situations, and selective activities for mentalizing were not observed. Selective brain activities for mentalizing became observable as a child’s age increased (Gweon et al., 2012). This may represent a “specialization” of the mentalizing regions that bear the social cognitive function. The series of studies described here suggests that this specialization is immature even in preschool children who could pass the Sally-Anne task, a brain function specific to mentalizing begins to form in the school-age period, and selective brain activities related to this function are observed in the latter half of the school-age period and beyond.

Although the specialization of this function progresses in the school-age period and beyond, adult-like efficient information processing has not yet been established during this period. For example, using cartoons, Wang et al. (2006) measured brain activity with fMRI when 9–14-year-old children and adults were asked to judge whether a character was being sincere or ironic, by speculating about the intention of the character. They found that the groups had no difficulty distinguishing irony from sincerity, and the activity in the medial frontal cortex increased in both the children and adults in the irony condition. However, the activity in this brain region increased more in the children compared with the adults (Wang et al., 2006). The observation that children require more brain activity than adults to perform a social cognitive task was also reported in a study of 12–18-year-old children (Blakemore et al., 2007).

In general, when the brain repeats a certain experience for a long time, it becomes unnecessary to fully recruit the activity in the particular brain regions that are used during the experience. For example, one study showed that a top-level soccer player could move his foot by recruiting a smaller amount of brain activity (BOLD signal) in the foot section of the M1 (Naito and Hirose, 2014). A similar phenomenon has been reported in musicians’ brains (e.g., Krings et al., 2000). Thus, one may associate the recruitment of less motor activity to generate a well-practiced movement with efficient neural control of that movement. Here, we also raise the possibility that the smaller increase in the BOLD signal could be related to a reduction of synaptic activity due to enhanced synaptic efficacy in M1 through extended motor practice, as shown in a non-human primate study (Picard et al., 2013). We think that social cognitive function is no exception. The mentalizing function is also believed to be efficiently conducted through the repeated experiences of speculating on the psychological states of other persons. In the brains of early teens who have little experience with mentalizing, this efficiency has not yet been attained. It is then possible to speculate that excessive brain activity in the prefrontal cortex would be measured in these children due to less experience mentalizing compared with adults (Wang et al., 2006; Blakemore et al., 2007).

This interpretation is not inconsistent with the fact that the structure of the prefrontal cortex is still in an immature state of development. As discussed in Section “Developmental Changes Observed in Brain Structure”, it takes a rather long time, or approximately 15–20 years, for the prefrontal cortex to structurally mature to the adult level. More specifically, excessive synapses are present in the early teens, and these synapses are pruned during the latter half of the teens while the minimally required synapses are preserved. It is conceivable that the brain acquires efficient information processing circuits for mentalizing (social cognitive) function in the prefrontal cortex through the synaptic pruning during this period.

## Development of the Brain Observed in Network Changes

Neuroimaging techniques have been mainly used to delineate the functions of various parts of the brain. Recently, however, the dominant view posits that a brain function is mainly implemented in a network, taking into consideration interactions between different brain regions, since it is most likely that individual brain regions do not function independently; rather, the brain realizes its specific functions by exchanging information between multiple regions. This viewpoint has been considered important in understanding the development of brain functions, and thus, the analysis of brain networks has been introduced in imaging studies in children. In this section, we introduce recent studies that measure resting-state brain activity while infants and children are not performing any particular task (see “Introduction: The Field of Developmental Cognitive Neuroscience” Section).

Fransson et al. (2007, 2009) examined resting-state connectivity during sleep in neonates using fMRI and reported six resting-state networks. These include the parieto-cerebellar network, the medio-lateral prefrontal network, and the bilateral basal ganglia network, in addition to the visual, sensorimotor, and auditory networks, similar to the adult networks. However, the cortical midline network (the default-mode network [DMN]: where activity is higher when at rest than when performing a particular task; Raichle et al., 2001) was not readily apparent in the neonate brain. Indeed, when the same researchers further examined cortical hubs in the neonates, they showed that the majority of hubs are located in the visual, sensorimotor, and auditory networks, whereas many hubs are located in the DMN in adults (Fransson et al., 2011). Functional connectivity networks (at least their precursors) seem to rapidly develop during the first year of life (infancy). Indeed, Gao et al. (2016) recently demonstrated the development of nine functional connectivity networks that include the DMN during infancy.

In children, de Bie et al. (2012) measured resting-state functional connectivity with fMRI in 5–8-year-old awake children and identified 14 components. They reported that among these networks, those for basic motor function and sensory-related processing seemed to have a functional organization similar to mature adult patterns. In contrast, the DMN and other networks involving higher-order cognitive functions exhibited immature characteristics. Furthermore, Fair et al. (2009) measured resting-state functional connectivity with fMRI in 7–9-year-old children, 10–15-year-old children and adults and showed the possibility that functional maturation is driven both by the segregation of nearby functional areas and by the integration of distant regions into a functional network. Interestingly, they found that adult-like functional integration in the DMN is still immature in the 10–15-year-old children. If we consider the fact that the adult DMN is usually formed by the medial prefrontal cortices, the medial parietal cortices, the temporal parietal junction, and the temporal cortices, corresponding to the social brain network (Mars et al., 2012; Takahashi et al., 2014), the slower DMN maturation might be associated with the slower development of social cognitive function described above.

We should also point out the possibility that the process of forming a functional network continues for a relatively long time even in the basic brain system for motor function. For example, a functional network in the M1 continues to change even during the school-age period. Nebel et al. (2014) measured the resting-state brain activity in 8–12-year-old children and adults with fMRI. They examined the somatotopic organization of the M1 and found that body representations for the upper and lower limbs were differentiated in the 11- and 12-year-old children as in adults; however, in the 8- and 9-year-old children, this differentiation was not observed (Nebel et al., 2014). Thus, it is conceivable that, in children younger than 10 years of age, somatotopic organization in M1 is still in the process of maturation.

The technical advances in network analysis have reached a level that enables the estimation of brain age by analyzing the connectivity of the brain network (Dosenbach et al., 2010). Dosenbach et al. (2010) measured resting-state brain activity for several minutes by fMRI in participants ranging in age from 7 to 30 years old and performed a pattern classification analysis using the data obtained from as many as 160 brain regions. They found that the strength of functional connectivity among remote brain regions tended to increase with increasing age and that the strength of functional connectivity among close brain regions tended to decrease with increasing age. By using this type of pattern analysis, the brain age can be estimated. From the viewpoint of functional connectivity of the brain, the authors suggested that the maturation of the brain appears to continue until approximately the age of 22. However, it is still unknown whether developmental change in functional connectivity is related to structural changes, such as myelination and synaptic pruning. This is an interesting and important issue to be solved in terms of plasticity in the brain.

Finally, advances in brain network research are not only clarifying the route to typical development, but also approaching an understanding of brains with atypical development. There has been an increasing number of studies (see below) on the network analyses of the brains of individuals with autism spectrum disorders (ASD), which are characterized by persistent deficits in social communication and social interaction, in conjunction with restricted, repetitive patterns of behavior, interests, or activities (Diagnostic and Statistical Manual of Mental Disorders, DSM-5, American Psychiatric Association, 2013). The network analysis was introduced because the pathology of ASD was not fully explained by a disorder in a specific part of the brain, and evidence is accumulating to show that abnormalities in network connectivity better explain the pathology of ASD.

Indeed, the recent view is that functional connectivity between remote brain regions is weak in individuals with ASD (Schipul et al., 2011; Vissers et al., 2012). In particular, the functional connectivity between the medial prefrontal cortex and the posterior cingulate gyrus that constitutes the DMN is weaker in individuals with ASD than in neurotypical individuals, and this connectivity has been reported to be correlated with the degree of social disorders (Weng et al., 2010; Jung et al., 2014). At the same time, the functional connectivity among nearby brain regions seems to increase in individuals with ASD, although the body of evidence is still limited (Courchesne and Pierce, 2005; Maximo et al., 2013). The characteristic functional connectivity in individuals with ASD is also found in the basic motor system. Namely, the less differentiated somatotopical representations in M1 are still observable in the 11- and 12-year-old children with ASD (see above and Nebel et al., 2014).

As discussed above, the pathology of ASD, which could not be clarified by functional mapping studies alone, is becoming clear by understanding functional networks. In the future, we hope that these findings will advance studies in the early diagnosis of ASD and will support individuals with ASD.

## Conclusion

In this article, we have described the changes that the brain undergoes both structurally and functionally over approximately the 20 years after birth. In neuroimaging studies in children, brain activities are often measured based on behavioral findings already obtained in the field of developmental psychology. However, developmental changes in the brain do not necessarily correspond to changes at the behavioral level. Even when similar behaviors are observed, the ways of processing that generate the behaviors can be varied. Indeed, the review of previous studies indicates that there seems to be a significant difference between how children use the brain and how adults use the brain, even though no significant differences are observed at the behavioral level. Normal perceptual, cognitive, and motor functions are acquired in and around the school-age period, but the specialization and the efficient information processing of these functions have yet to mature in the brain. In other words, the true meaning of brain development can only be understood by examining the brains of children structurally, functionally and at the network level.

Furthermore, there is more to developmental cognitive neuroscience than understanding human development. The most notable characteristic of the brain is its plasticity; in modern brain science, it has become commonly accepted that even the injured brain of an adult is expected to recover its functions by plasticity. Understanding how neural circuits are formed in the developmental stages of the brain and how pruning and functional inhibition are accomplished to realize a certain function should shed light on the strategy that the brain uses in order to restore damaged functions after injury. Finally, to understand the brain correctly is to understand humans correctly. Developmental cognitive neuroscience using neuroimaging technology contributes to the correct understanding of human beings.

## Author Contributions

All authors (TM, MA, and EN) discussed the material presented in this review, wrote the manuscript, and approved the final version of the manuscript for submission.

## Conflict of Interest Statement

The authors declare that the research was conducted in the absence of any commercial or financial relationships that could be construed as a potential conflict of interest.
